# Moisture legacy effects shape vegetation productivity across East Asia ecosystems, 2001-2020

**DOI:** 10.3389/fpls.2025.1649987

**Published:** 2025-08-06

**Authors:** Pingping Zheng, Mark Henderson, Binhui Liu, Mingyang Chen, Kexin Deng, Ruiting Gu, Xiaojing Gong

**Affiliations:** ^1^ College of Forestry, The Northeast Forestry University, Harbin, China; ^2^ Mills College, Northeastern University, Oakland, CA, United States; ^3^ Changzhou Architectural Research Institute Group Co., Ltd., Changzhou, China

**Keywords:** gross primary productivity, vegetation phenology, climate change, relative humidity, soil moisture

## Abstract

This study investigated spatiotemporal patterns of growing season gross primary productivity (GPP_GS_) across three vegetation types in mid-to-high latitude East Asia from 2001-2020.Growing season parameters and GPP were extracted from MODIS satellite data and combined with meteorological data to examine climate-vegetation relationships through trend analysis and correlation methods. GPP_GS_ increased significantly overall (4.12 gC/m2/yr), with deciduous broad-leaved forest (DBF) having highest productivity (1035.52 gC/m2), followed by deciduous needle-leaved forest (DNF) (830.83 gC/m2) and grassland (800.62 gC/m2). A critical divergence occurred around 2014, when grassland and DNF growth rates declined substantially while DBF maintained steady increases. Phenological factors showed limited explanatory power for GPP variations, albeit GPP are sensitive to vegetation peak growth time for all three vegetation types. Climate analysis identified relative humidity (RH) as the dominant driver, with the previous year’s growing season RH showing around 35.91% stronger positive correlations than current year values across all vegetation types; the difference is highest in DNF and the least in grassland. We conclude that the legacy effects of atmospheric moisture conditions explained the 2014 divergence, highlighting the increasing importance of water availability under global warming. Increases in atmospheric dryness accompanied by temperature increases will affect vegetation carbon storage and the societal-economic services provided by these ecosystems.

## 1 Introduction

Gross Primary Productivity (GPP) of terrestrial ecosystems refers to the amount of organic matter fixed by green plants per unit area per unit time through photosynthesis and a series of internal physiological processes (Anav et al., 2015; Campbell et al., 2017; Hilty et al., 2021). GPP is directly related to the strength of the carbon sink of terrestrial vegetation and represents the largest carbon flux between terrestrial ecosystems and the atmosphere (Zhao et al., 2005; Beer et al., 2010; Hu et al., 2018).

Extensive research has confirmed that terrestrial plant ecosystems in mid- to high-latitude regions play a crucial role in regulating the global carbon cycle and atmospheric CO_2_ concentrations (Dang et al., 2023). In East Asia, these latitudes have seen rapid warming in recent decades and are home to vegetation with high sensitivity to climate change (Lv et al., 2023; Chen et al., 2024b). Studying the pattern of change in GPP and its connection with the changing climate will help to improve our understanding of the carbon cycle.

While most of the previous studies qualitatively or quantitatively analyzed the direct influence of either meteorological factors or phenology on the annual GPP dynamics (Wang et al., 2021; Wu et al., 2021), fewer studies have investigated combined the influence of climate and vegetation phenology simultaneously on the magnitude of changes in vegetation productivity (Fu et al., 2018; Xu et al., 2021). In addition, current research on the relationship between vegetation growth and climatic factors focuses mainly on climatic factors such as temperature and precipitation (Hu et al., 2021; Liao et al., 2024), and most of them ignoring the possible differences in the response of different vegetation types. There is a need for studies to simultaneously investigate the combined influence of climate and vegetation phenology on changes in vegetation productivity and to consider the possible differences in response among different vegetation types. Therefore, for this study we use MOD17A2HGF.061 GPP data from 2001–2020 along with meteorological data to investigate the spatial and temporal characteristics of growing season cumulative gross primary productivity (GPP_GS_) of different vegetation types and their responses to climate change and vegetation phenology in the middle to high latitudes of East Asia.

### 1.1 Factors influencing GPP

Spatiotemporal variations in global terrestrial GPP are related to climatic conditions, Photosynthetically Active Radiation (PAR, as a source of energy for photosynthesis), vegetation types, and land use patterns (Ichii et al., 2005; Ahlström et al., 2015; Yao et al., 2018; Xie et al., 2021; Chen et al., 2022). Among climatic factors, temperature and precipitation have been shown to be critical in the dynamics of vegetation GPP (Chen et al., 2021; Meng et al., 2023b; Xu et al., 2023).They impact carbon absorption directly by altering photosynthetic rates and water use efficiency, or indirectly through modifying the rhythm of plant phenology (Hatfield and Dold, 2019; Shi et al., 2022; Xiao et al., 2023).

Other climate indicators such as air and soil moisture conditions also play important roles. Soil moisture (SM) determines the amount of water that can be drawn by plant roots, and its fluctuations can affect leaf structure and root growth, thereby influencing vegetation photosynthesis (Green et al., 2019; Liu et al., 2020b; Xu et al., 2024). In terms of air moisture conditions, elevated vapor pressure deficit (VPD) significantly reduces stomatal conductance and limits the actual photosynthetic rate, thereby reducing vegetation productivity (López et al., 2021). Similarly, excessively low relative humidity (RH) causes the closure of plant leaf stomata and a reduction in the amount of carbon dioxide available to the plant, which in turn leads to a reduction in the efficiency of photosynthetic carbon fixation (Zhao et al., 2019; López et al., 2021). In either case, a shortage of water can limit plant growth and ecosystem carbon fluxes, but there are some differences and commonalities between the effects of VPD and RH. Firstly, it has been found that climate warming has a minimal effect on RH, but VPD is increasing exponentially, suggesting that VPD and RH do not change synchronously (Held and Soden, 2000). Secondly, VPD reflects the combined changes in air temperature and relative humidity: high temperatures and stable RH lead to higher VPD, which increases plant water demand and reduces carbon assimilation rate (Zhang et al., 2021). Compared with RH, VPD more directly impacts the water use efficiency of trees, thereby affecting vegetation productivity (Sato et al., 2015; Li et al., 2023). With global warming, if water vapor pressure increases at a proportional rate with saturated water vapor pressure (which is temperature-dominated), the VPD will increase, but RH more likely remain stable. Therefore, VPD may not change in synchrony with RH, with RH lagging behind VPD.

GPP is the cumulative rate of gross plant photosynthesis over time. Over the course of the growing season and from year to year, changes in vegetation phenology may induce GPP changes (Xia et al., 2015). In general, vegetation phenology directly affects surface energy exchange, water cycle, and terrestrial carbon cycle,and plays a critical role in determining the duration of canopy photosynthetic activity, and drives ecosystem carbon sequestration (Richardson et al., 2010; Wang et al., 2017). As such, climate change not only affects GPP directly, but may also modulate GPP through altering phenology (Baldocchi et al., 2018). In addition, the effects on vegetation productivity through climate change and phenology changes may vary across different regions or vegetation types (Abbas et al., 2021). For example, the relative impacts of climate change and phenological changes on grassland productivity in the Tibetan Plateau vary with grassland type (Xiao et al., 2023). An earlier start to the growing season (SOS) in spring and higher photosynthetic peaks in summer was found to alter the seasonal pattern of vegetation productivity in the middle and lower reaches of the Yangtze River, China (Yang et al., 2024). A recent study also demonstrated that, in the mid- to high-latitude regions of the Northern Hemisphere, early SOS and a delayed end of the growing season (EOS) contributed to the increase in forest GPP in spring and autumn, respectively (Wang et al., 2025). These findings confirm that changes in phenology, serving as a sensitive and direct indicator of climate change, also influence the seasonal and annual dynamics of GPP (White et al., 2009), and that the effects of phenology and climatic factors, while not completely synchronized, play direct or indirect roles in the variation of GPP (Yang et al., 2017).

### 1.2 Study objectives

The objectives of this study include (1) investigating the spatial and temporal variation of GPP_GS_ for different vegetation types in the mid-to-high latitude of East Asia; (2) analyzing the relationships between GPP_GS_ and growing season climatic variables—maximum temperature (T_max_), minimum temperature (T_min_), photosynthetically active radiation (PAR), precipitation (P), relative humidity (RH), soil moisture (SM), and Vapor Pressure Deficit (VPD)—as well as phenological variables; and (3) determining the dominant factors driving temporal changes in GPP_GS_ among climate and phenological indicators for grassland, deciduous needleleaf forests, and deciduous broadleaf forests during the period of 2001-2020. By elucidating the intricate relationship between vegetation GPP and climate change in this region, our findings offer valuable insights for predicting shifts in vegetation productivity under future global warming scenarios.

## 2 Materials and methods

### 2.1 Study area

**Figure 1:**
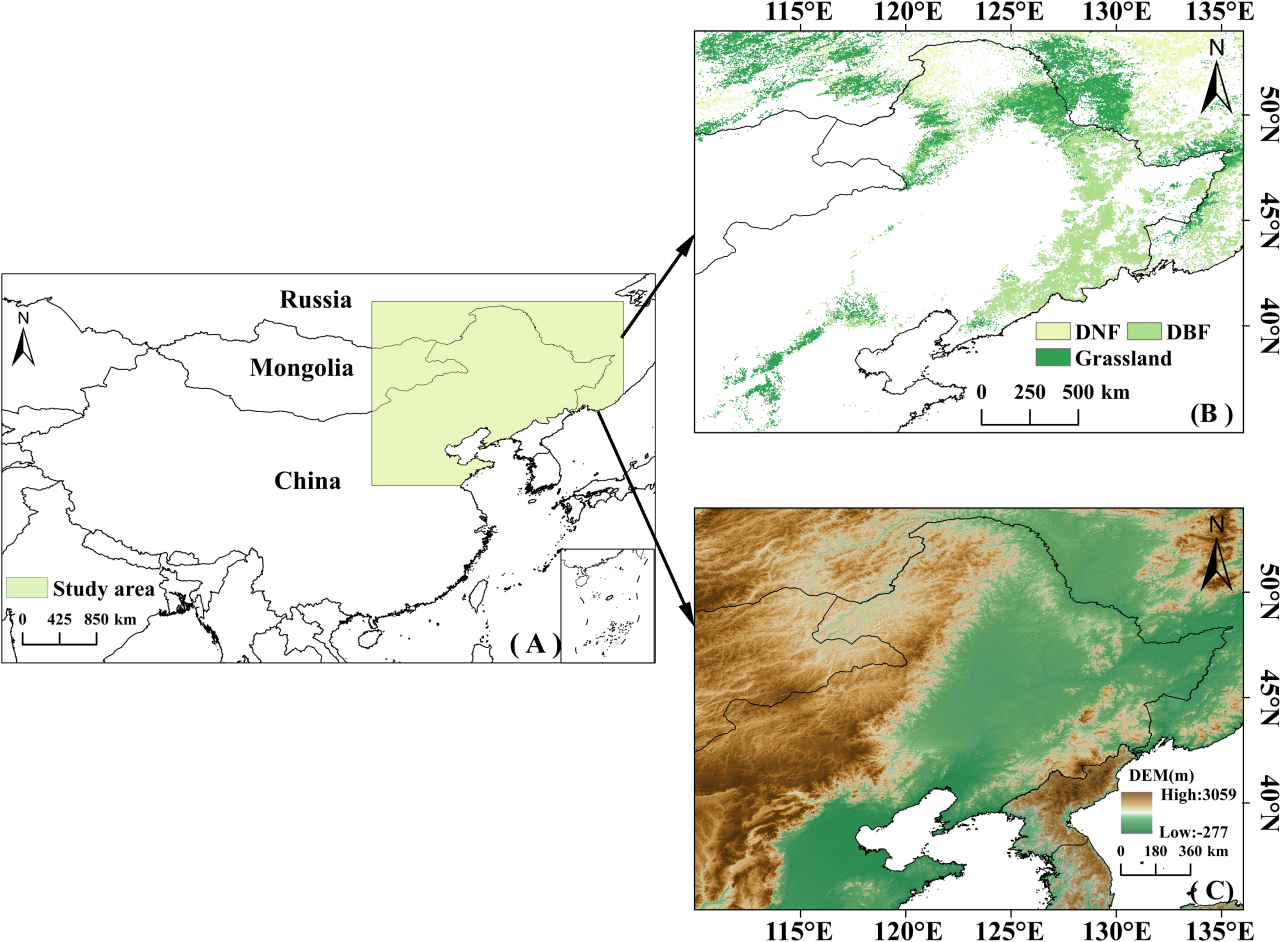
**(A)** Schematic map of the study area; **(B)** Distribution of the three vegetation types in the study area; **(C)** Elevation map of the study area.

The study area is located in the middle and high latitudes of northeastern Asia (35° to 54°N, 110° to 135°E), primarily including Northeast China and parts of Russia (**Figure 1A**). This region has experienced rapid temperature increases over the past few decades (IPCC, 2014). The topography is characterized by higher elevations in the west and lower in the east (**Figure 1C**). The majority of the study area belongs to the temperate continental climate zone, whereas the southeastern coastal areas are characterized by a temperate monsoon climate; precipitation and temperature increase from northwest to southeast. With complex topography and spatial variability in climate conditions, the study area’s vegetation is highly sensitive to climate change (Wang et al., 2022a; Chen et al., 2024b). The study area includes three vegetation types: grasslands in the north, south, and west; deciduous broadleaf forests (DBF) in the east; and deciduous needleleaf forests (DNF) in the north (**Figure 1B**). Excluding other land cover types such as agriculture, desert, or urban uses, or areas that changed during the study period, DBF, grasslands, and DNF make up 47.09%, 44.23%, and 8.68%, respectively, of the study area.

### 2.2 Datasets

#### 2.2.1 Phenology and GPP

This study utilized two Moderate Resolution Imaging Spectroradiometer (MODIS) products with a spatial resolution of 500m covering the years from 2001 to 2020. These products, released by the U.S. National Aeronautics and Space Administration (NASA) (https://www.earthdata.nasa.gov). included annual vegetation phenology data (MCD12Q2) and 8-day GPP data (MOD17A2HGF.061). The vegetation phenology products were derived from the time series of Normalized Difference Vegetation Index (NDVI). Four phenological indicators were selected for analysis: SOS, the start of the growing season; POS, the time of the peak growing season; EOS, the end of the growing season; and LOS, the length of the growing season. Additionally, the 8-day GPP were converted to months, and the cumulative GPP for the growing season of each year (April-October) was computed (hereafter denoted as GPP_GS_) to obtain the 20-year GPP_GS_ time series (Hu et al., 2021).

#### 2.2.2 Meteorological data

Meteorological data were obtained from the climate dataset produced by the National Tibetan Plateau Data Center (https://data.tpdc.ac.cn/), with a spatial resolution of 1 km covering the period from 1901 to 2023. From this dataset, we obtained climate data include maximum temperature (T_max_), minimum temperature (T_min_), soil moisture (SM) and precipitation (P). Photosynthetically Active Radiation (PAR) and Relative Humidity (RH) were obtained from the National Earth System Science Data Centre (https://www.geodata.cn/main/), with the PAR dataset spanning the period of 2000–2022 with a spatial resolution of 0.05°, whereas the RH dataset covers the period of 2000–2020 with a spatial resolution of 1 km. We obtained all meteorological data on a monthly scale for the period 2001–2020 and calculated the growing season (April-October) average, except for precipitation, which was calculated as the cumulative value for the growing season.

To obtain unified resolution, we resampled the meteorological data to a 500m resolution, consistent with the GPP data resolution, using a NEAREST interpolation method commonly adopted by prior studies (Zu et al., 2018; Mei et al., 2021; Shen et al., 2024b).

#### 2.2.3 Other data

The MCD12Q1 dataset (https://ladsweb.modaps.eosdis.nasa.gov) provides the annual distribution of different land cover types globally at 500 m spatial resolution. The land cover types in the study area were classified into DBF, DNF and grassland by taking the areas that did not change between 2001 and 2020 (**Figure 1B**). The digital elevation model (DEM) data were obtained from the geospatial data cloud (https://www.gscloud.cn/search), with a resolution of 90 m.

### 2.3 Methods

#### 2.3.1 Extraction of vegetation phenology parameters

In this study, the dynamic thresholding method of S-G filtering method was used to extract vegetation phenology parameters using TIMESAT in MATLAB software. Firstly, in order to remove the random noise in the time series, the MODIS data were smoothed by S-G filtering, which has been widely used for data smoothing and noise reduction as it eliminates the noise while maintaining the shape and width of the original signal (Jeong et al., 2011). Next, the dynamic thresholding method was used to extract vegetation phenology parameters (Jonsson and Eklundh, 2002). This method is based on the relative amplitude of the whole time series to obtain the vegetation phenology period information. According to the criteria of former related research, define the start of the growing season (SOS) and the end of the growing season (EOS) as the dates of the first crossing the 30% NDVI amplitude and the last crossing the 50% NDVI amplitude, respectively (Piao et al., 2020). The length of the growing season (LOS) was obtained by subtracting the end date of the growing season from the start date of the growing season. The time when NDVI reaches its maximum amplitude marks the peak of vegetation growth (POS), representing the date on which vegetation photosynthesis reaches its maximum (Liu et al., 2025). The dynamic threshold model is as follows (Myneni et al., 1997; Sisheber et al., 2022):


$${\text{NDVI}}_{\text{thr}}=\left({\text{NDVI}}_{\text{max }}-{\text{NDVI}}_{\text{min}}\right)\times \text{C}$$

where NDVI_thr_ is the threshold value, NDVI_max_ is the maximum value in the ascending phase, NDVI_min_ is the minimum value in the decreasing phase, and C is the coefficient.

#### 2.3.2 Trend analysis

To detect temporal trends of climatic variables, vegetation phenology, and annual GPP over the past 20 years and their corresponding significance, the non-parametric Sen’s slope method and Mann-Kendall trend analysis were used (Sen, 1968). We analysed the data on a raster scale using Matlab R2024a software. The slope was calculated by the following equation:


$$Slope=\text{Median}\left[\frac{X_{j}-X_{i}}{j-i}\right],\forall \;1\text{≤i}\leq \text{j}\leq \text{n}$$

where x_j_ and x_i_ represent the values at times i and j, and n represents the length of the time series data. When Slope is positive, it indicates that the variable has an upward trend; when Slope is negative, it indicates that the variable has a downward trend; the larger the absolute value of Slope, the larger the magnitude of the change trend. An upward slope suggests a delay in the phenological period and an increase in GPP accumulation and climate factors. Conversely, a downward slope suggests an advancement in the phenological period and a decrease in GPP accumulation and climate factors.

To analyze temporal variation, we applied a nine-point binomial filter with reflected ends to smooth out year-to-year variations and show the longer-term trend.

#### 2.3.3 Correlation analysis

We calculated the correlation between phenological and climatic factors and GPP_GS_ using correlation analysis. The correlation coefficient r was calculated by the following equation:


$$r=\frac{\sum_{i=1}^{n}\left(x_{i}-\bar{x}\right)\left(y_{i}-\bar{y}\right)}{\sqrt{\sum_{i=1}^{n}{\left(x_{i}-\bar{x}\right)}^{2}}\sqrt{\sum_{i=1}^{n}{\left(y_{i}-\bar{y}\right)}^{2}}}$$

where r is the correlation coefficient of the two variables, $\bar{\text{X}}$ and $\bar{\text{Y}}$ are the average of the two variables over the time series and n is the number of years. In addition, the t-test was used to test the significance of the correlation (Zhang et al., 2023).

## 3 Results

### 3.1 Spatiotemporal distribution and change of GPP_GS_

**Figure 2:**
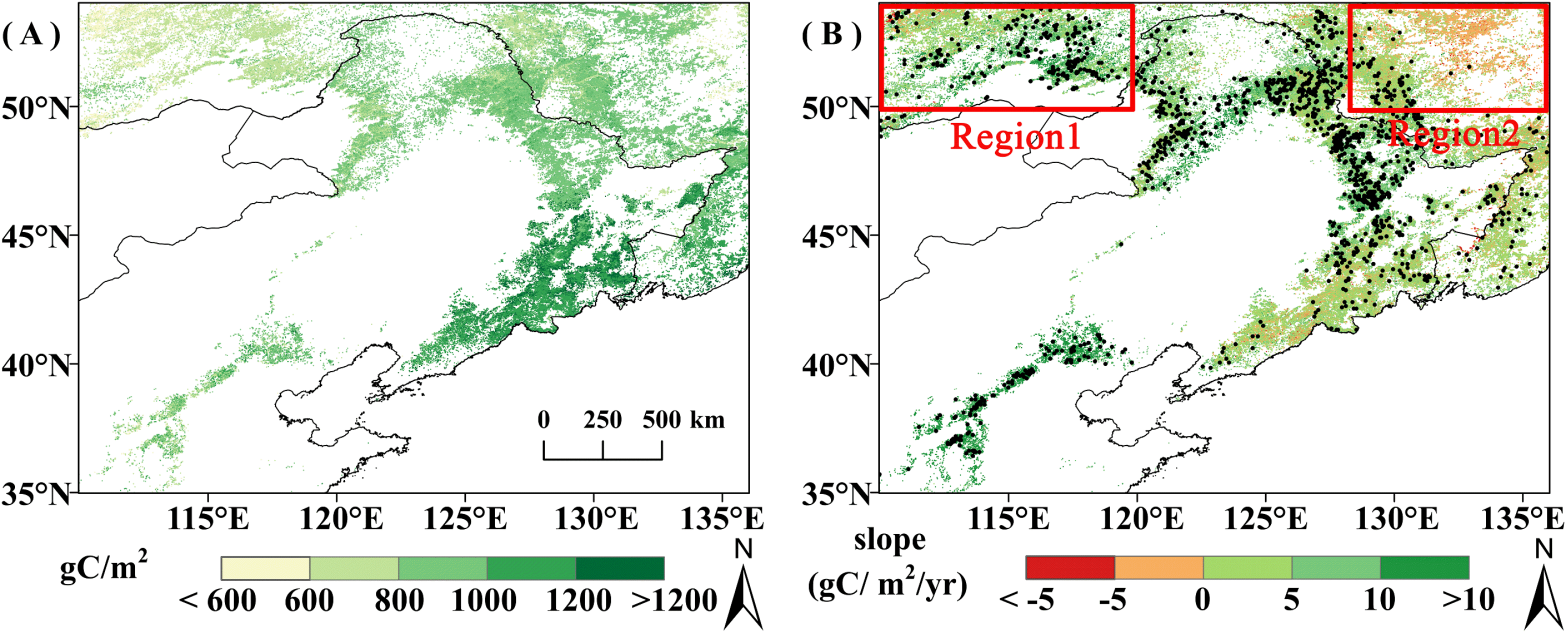
**(A)** Spatial distribution of average GPP_GS_ from 2001 to 2020; **(B)** Spatial trend of GPP_GS_ from 2001 to 2020, pixel points with dots indicate significant trends (*P*<0.05).

Spatially, average GPP_GS_ increased from northwest to southeast (**Figure 2A**). This is consistent with the spatial distribution of vegetation types and climate. Precipitation and temperature during the growing season increase from northwest to southeast, and the higher GPP_GS_ areas were basically distributed in the deciduous broad-leaved forest distribution area with better temperature and moisture conditions, while the lower GPP_GS_ areas were distributed in the grassland and deciduous coniferous forest distribution area with relatively poor temperature and moisture conditions.

**Figure 3:**
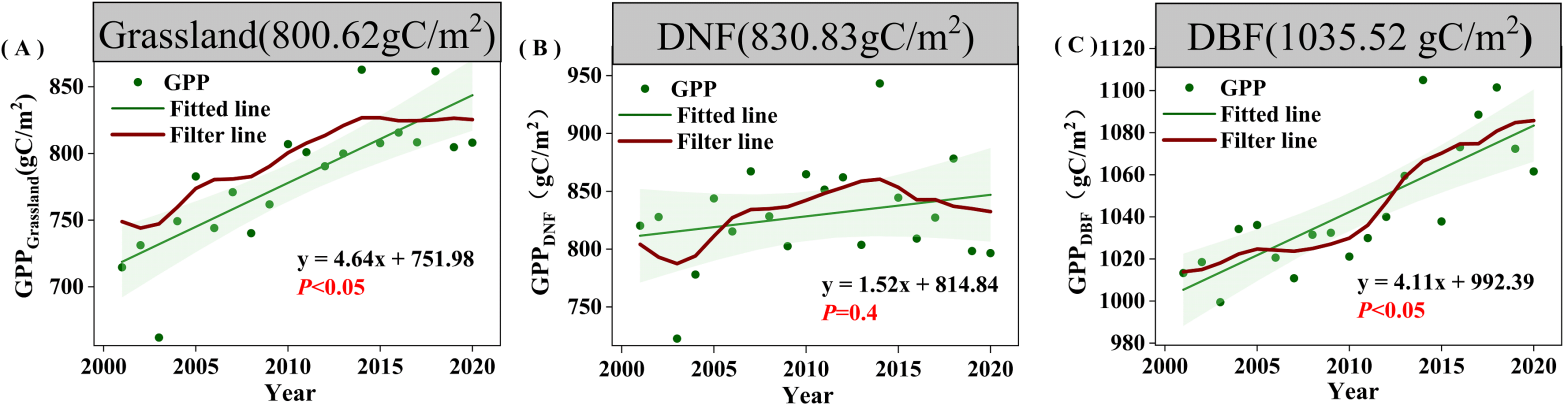
Changes in GPP_GS_ for different vegetation types from 2000 to 2020.**(A)** Grassland. **(B)** DNF. **(C)** DBF. The red line represents the result of nine-point binomial filter with reflected ends; the green line represents the trend line; and the numbers in the grey boxes represent the mean GPP_GS_ of the corresponding vegetation types.

The overall average vegetation GPP_GS_ was 913.81 gC/m^2^, with DBF having the highest average GPP_GS_ (1035.52 gC/m^2^), followed by DNF (830.83 gC/m^2^), and grassland (800.62 gC/m^2^) (**Figure 3**). The trend of GPP from 2001 to 2020 is shown in **Figure 2B**. The area of showing an increasing trend accounted for 85.46% of the study area, of which 33.29% increased significantly (*P*<0.05). The areas of DBF and grassland GPP_GS_ with an increasing trend is obviously more than the area with decreasing trend: increases are seen in 88.58% of DBF area (39.54% significant) and 88.62% of grassland area (39.32% significant). The area of DNF GPP_GS_ with an increasing trend (50.47%) was basically the same as the area with a decreasing trend (49.49%), but the much more of the increasing trend areas was significant (31.46% compared with 0.80%). Increases in DNF GPP_GS_ are mainly located in the relatively arid northwest corner of the study area (**Figure 2B**, Region 1), while decreases in DNF GPP_GS_ are primarily found in the relatively humid northeast corner of the study area (**Figure 2B**, Region 2). That is, for DNF, spatial variation in the trend is connected with background moisture conditions. The area of significant decline in grassland GPP_GS_ is mainly distributed in the eastern part of the study area, and there is no obvious spatial difference in the DBF trend.

The overall trend of vegetation GPP_GS_ in the study area from 2001 to 2020 shows a significant increase (4.12gC/m^2^/yr, *P*<0.05). Grassland and DBF GPP_GS_ showed significant increasing trends at similar magnitudes of 4.64gC/m^2^/yr and 4.11gC/m^2^/yr ^(^ *P*<0.05), respectively; while DNF GPP_GS_ showed a non-significant increasing trend with the smallest magnitude of 1.52gC/m^2^/yr (P=0.4) (**Figure 3**). For DNF, relatively arid Region 1 had a significant increase in GPP_GS_ (6.68gC/m^2^/yr, *P*<0.05), and relatively humid Region 2 had a non-significant decreasing trend (-0.43gC/m^2^/yr, *P*=0.85).

As can be seen from **Figure 3**, DBF GPP_GS_ in the study area showed a continuous increase from 2001 to 2020, while DNF and grassland GPP_GS_ had a similar trends, i.e., both diverged around 2014. Among them, grassland showed a significant increasing trend before 2014, at 7.42 gC/m^2^ per year (*P*<0.05), with growth leveling off after 2014, while DNF GPP_GS_ also showed a significant upward trend before 2014, with a trend of 6.79gC/m^2^ per year (*P*<0.05), and then showed a slight decline after 2014. Compared with the trend during 2001-2014, the increase rates of DBF, grassland, and DNF GPP_GS_ were 6%, 37.44%, and 77.59% lower, respectively, than the trend during the period between 2001 and 2020.

In summary, the interannual variation in GPP_GS_ of DNF and grassland diverged around 2014. In order to further understand the reasons for the different temporal change character in GPP_GS_ of different vegetation types, we first examined the sensitivity and the relationship between changes in GPP_GS_ and changes in phenology of different vegetation types.

### 3.2 GPP_GS_ phenology sensitivity

The correlations between GPP_GS_ and phenological indicators showed some significant relationships (**Table 1**). The common feature was that all vegetation types had a significant negative correlations between GPP_GS_ and POS, while the overall correlation with EOS was weak and insignificant, indicating that EOS was not the main factor affecting vegetation gross primary productivity. Meanwhile, grassland and DBF showed significant negative correlations between GPP_GS_ and SOS and significant positive correlations with LOS. For DNF, there was a significant negative correlation of GPP_GS_ with POS in the relatively humid area (Region 2), while in the relatively arid area (Region 1) there was a significant negative correlation with SOS and a significant positive correlation with LOS, but with no significant correlation with POS.

**Table 1:** Correlation coefficients (r) between GPP_GS_ and phenological indicators of different vegetation types.

Region/Type	SOS	POS	EOS	LOS
Grassland	-0.40*	-0.71***	-0.05	0.39*
DBF	-0.52**	-0.42*	0.02	0.55**
DNF	-0.19	-0.43*	-0.04	0.12
Region1	-0.48**	-0.19	0.35	0.64***
Region2	-0.03	-0.41*	-0.26	-0.15

*Significant at the 0.1 level; **Significant at the 0.05 level; ***Significant at the 0.01 level. Region1 represent DNF located in the relatively arid area, Region2 represent DNF located in the relatively humid area.

During 2001-2020, vegetation GPP_GS_ was negatively correlated with SOS in 71.90% of the study area and with and POS in 71.80% of the study area; the correlations were statistically significant for 19.08% and 19.20% of the area, respectively. GPP_GS_ was positively correlated with LOS in 66.96% of the study area (significant in 18.29%). The percentages with positive and negative correlations with EOS (47.12% and 52.88%) did not differ much, with neither direction achieving statistical significance for more than 10% of the area.

**Figure 4:**
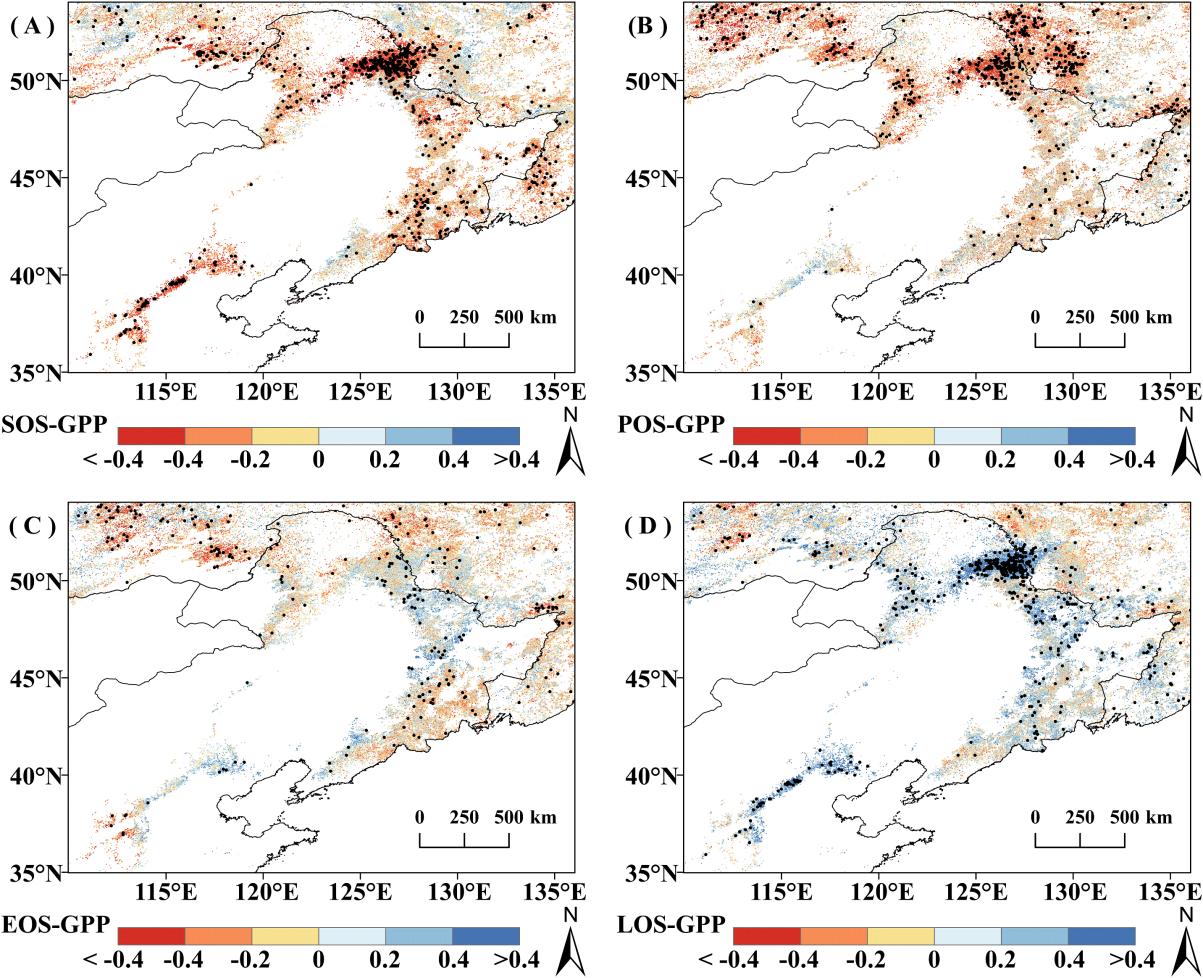
Correlation analysis between growing season cumulative productivity (GPPGS) and phenological factors from 2001 to 2020. **(A)** SOS. **(B)** POS. **(C)** EOS. **(D)** LOS , pixel points with dots indicate significant correlations (P<0.05).

In terms of different vegetation types, unlike the significant correlation between regional mean DNF GPP_GS_ and POS, the percentage of DNF area with GPP_GS_ significantly correlated with any of the phenological factors was relatively small (none of them was more than 10%). Grassland GPP_GS_ was more affected by POS, with 83.96% of pixels negatively correlated with POS (among which 28.22% were significantly correlated, *P*<0.05). DBF GPP_GS_ was relatively more affected by SOS, with 77.5% of pixels negatively correlated with SOS (15.19% significant, *P*<0.05), which was approximately the same as that of the correlation between the regional average GPP_GS_ and phenological factors (**Figure 4**).

**Figure 5:**
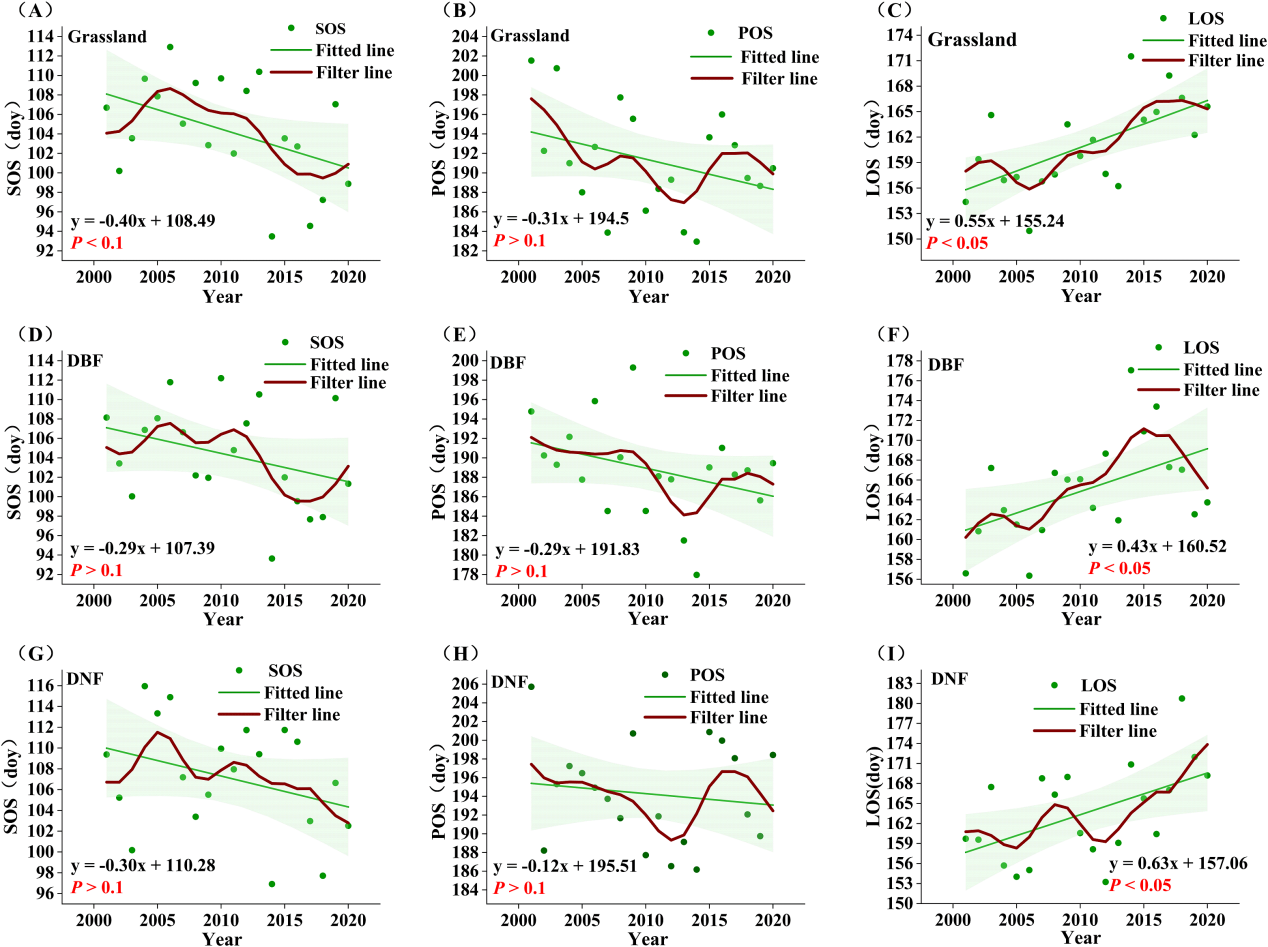
Annual average phenological indicators from 2001 to 2020. **(A-C)**: grassland SOS, POS, LOS; **(D-F )**: DBF SOS, POS, LOS; **(G-I)** : DNF SOS, POS, LOS. The red line represents the result of nine-point binomial filter with reflected ends; the green line represents the trend line.

According to the correlation results, SOS, POS, and LOS may be the phenological factors that cause changes in GPP_GS_. In order to compare the temporal changes of phenology and GPP_GS_, we use a nine-point binomial filter to smooth out year-to-year variations and show the longer-term trends. POS showed a non-significant trend of advancement in all vegetation types, with a relatively small proportion of significant pixels. SOS was significantly advanced only in the grassland area with a trend of −0.40 day yr^-1^ (*P*<0.1) (**Figure 5**), while the DBF and DNF areas showed non-significant trends. LOS showed significant lengthening trends (*P*<0.05) in all vegetation types, with the most substantial increase in DNF (0.63 day yr^-1^), followed by grassland (0.55 day yr^-1^), and the smallest increase in DBF (0.43 day yr ^-1^). This suggests that the lengthening of LOS in grassland and DBF affects GPP_GS_ to some extent, and advance of SOS in grassland can enhance the growth of GPP_GS_.

Further comparing the temporal change of phenological factors and GPP_GS_ in each vegetation type, we found that SOS did not advance consistently in all vegetation types through 2014, but there was a pattern of advancement after 2014. This contrasts with the observed trend of significantly increasing GPP_GS_ in all vegetation types through 2014 and the divergence after 2014 in DNF and grassland.

Similarly, LOS would not appear to be the dominant cause of the divergence in GPP_GS_ starting in 2014. For DNF, LOS has been consistently increasing, clearly inconsistent with the pattern of decline in DNF GPP_GS_ after 2014. For grassland, LOS fluctuated upward before 2016 before leveling off, failing to support the pattern of steady increase in GPP_GS_ before 2014 and stagnation after 2014; the only spatially significant increase in LOS occurred in the central part of the grassland, which could not support the widespread significant increase in the trend of grassland GPP_GS_.

As seen in **Figure 5**, the post-2014 delay and then advance in POS is clearly inconsistent with concurrent changes in GPP_GS_, but the advancement of POS prior to 2014 is contributed to the rise of GPP_GS_ in all three vegetation types. However, the proportion of pixel points where POS was significantly advanced accounted for only 9%, and the proportion of pixel points that were significantly correlated with GPP in general accounted for only 19.20%, with POS significantly correlated with GPP in only 31.94% of the pixel points that significantly increased in GPP. Thus, while POS may have played a role before 2014, but was not the main factor contributing to the change in GPP.

The above results show that, in DBF and grassland, GPP_GS_ is most strongly correlated with SOS and LOS, while across all vegetation types, the advancement of POS corresponds to increases in GPP_GS_, especially for grassland. However, given the spatial and temporal characteristics of change of each of the phenological indicators, phenology changes are not likely to be the main drivers of the observed changes in GPP in the period of 2001-2020, so we further analyzed the relationship between climatic factors and GPP_GS_.

### 3.3 GPP_GS_ climate sensitivity

Among the correlations between GPP_GS_ and the growing season climatic indicators of different vegetation types (**Table 2**), the overall weak and nonsignificant correlation between GPP_GS_ and T_min_ was lower than the correlation between GPP_GS_ and T_max_ for all vegetation types, indicating that T_min_ was not the main factor influencing the vegetation GPP. For DNF, the correlations of GPP_GS_ with T_max_ and PAR was both significantly positive, while the correlations with P and SM was significantly negative. Unlike DNF GPP_GS,_ DBF GPP_GS_ was significantly positively correlated with P but not significantly correlated with T_max_ and PAR. In addition, DBF and grassland GPP_GS_ were both strongly and significantly positively correlated with RH and significantly and negatively correlated with VPD. This suggests that the ability of DNF to produce organic matter is limited by thermal conditions but inhibited by excessive moisture, while grassland and DBF are limited by water conditions. In addition, DNF in the relatively humid area (Region 2) was also significantly correlated with PAR and T_max_, and negatively correlated with SM and P, while DNF in the relatively arid area (Region 1) was not significantly correlated with climatic factors. This suggests that the DNF results are more reflective of the relatively humid area, which accounts for a greater number of pixels that of the relatively arid area.

**Table 2:** Correlation coefficients (r) between GPP_GS_ and growing season climate indicators of different vegetation types.

Region/Type	T_max_	T_min_	P	PAR	RH	VPD	SM
Grassland	0.31	0.14	-0.13	0.24	0.62***	-0.41*	-0.12
DBF	0.30	0.24	0.42*	-0.17	0.63***	-0.46**	0.27
DNF	0.70***	0.01	-0.47**	0.69***	-0.07	0.37	-0.75***
Region1	0.27	0.19	0.22	0.15	0.31	-0.03	-0.06
Region2	0.69***	0.01	-0.57***	0.77***	-0.10	0.38*	-0.65***

*Significant at the 0.1 level; **Significant at the 0.05 level; ***Significant at the 0.01 level. Region1 represent DNF located in the relatively arid area, Region2 represent DNF located in the relatively humid area.

**Figure 6:**
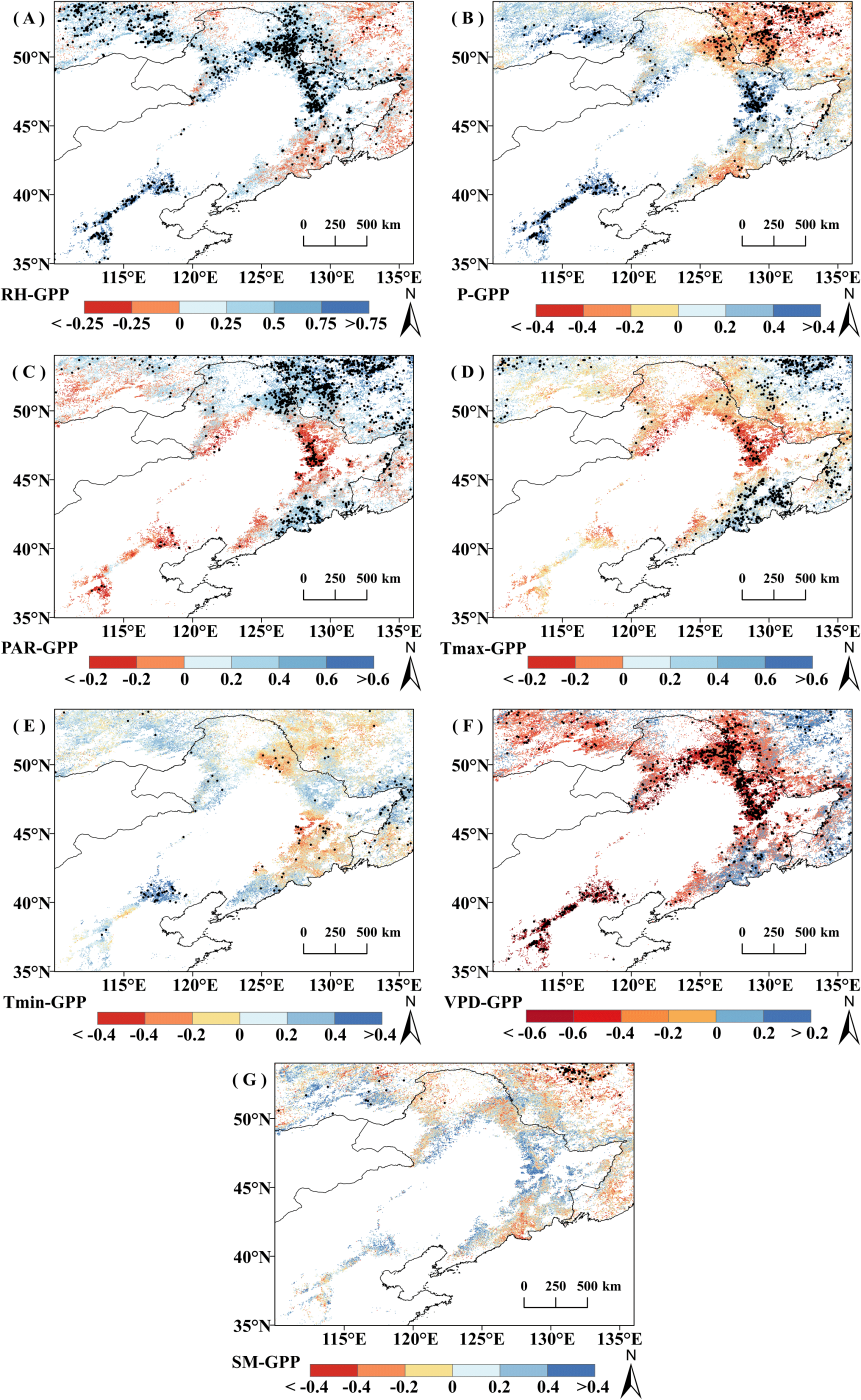
Correlation coefficient (r) between growing season cumulative productivity (GPP_GS_) and climatic factors from 2001 to 2020; pixel points with dots indicate significant correlations (P<0.05). **(A)** RH. **(B)** P. **(C)** PAR. **(D)** Tmax. **(E)** T min. **(F) **VPD. **(G) **SM.

The spatial character of correlation between GPP_GS_ and climate indicators is similar to those of the correlation between regional mean GPP_GS_ and climatic indicators (**Figure 6**). Overall, in 78.82% of the study area, vegetation GPP_GS_ was positively correlated with RH (of which 39.56% is significant, *P*<0.05). Vegetation GPP_GS_ was negatively correlated with PAR and T_max_ in the most of the study area, with 75.32% (26.27% significant) and 75.26% (20.14% significant) respectively. Vegetation GPP_GS_ was negatively correlated with VPD in 69.42% of the area (29.98% significant, *P*<0.05). The correlations between vegetation GPP_GS_ and SM or T_min_ did not reach a significant level over a large area, and the percentage of significantly correlated pixels did not exceed 15% (*P*<0.05).

In terms of different vegetation types, DBF and grassland GPP_GS_ were more affected by RH and VPD. The proportion of DBF pixels positively correlated with RH was 72.74% (34.46% significant, *P*<0.05) and those negatively correlated with VPD was 63.36% (33.79% significant, *P*<0.05), while the proportion of grassland GPP_GS_ pixels positively correlated with RH was 91% (47.05% significant, *P*<0.05) and those negatively correlated with VPD was 84.87% (39.19% is significant, *P*<0.05). DNF GPP_GS_ was most greatly affected by T_max_ and PAR, with 93.26% of pixels positively correlated with T_max_ (62.73% is significant, *P*<0.05) and 90.71% with PAR (73.78% significant, *P*<0.05).

It is worth noting that there was obvious spatial variability in the effects of P on GPP_GS_ in grassland and DNF, with a largely positive response to P distributed mainly in the relatively arid region and negative correlations mainly distributed in the humid region adjacent to the sea. Positive and negative correlations offset each other, resulting in a nonsignificant correlation of GPP_GS_ with P for grasslands and a significant negative correlation for DNF, which is concentrated in the relatively wet zone, Region 2. Similarly, DNF GPP_GS_ was significantly positively correlated with current year RH mainly in the northwestern part of the study area (Region 1), and negatively correlated with current year RH mainly in the relatively wet region in the northeastern part of the study area (Region 2).

**Figure 7:**
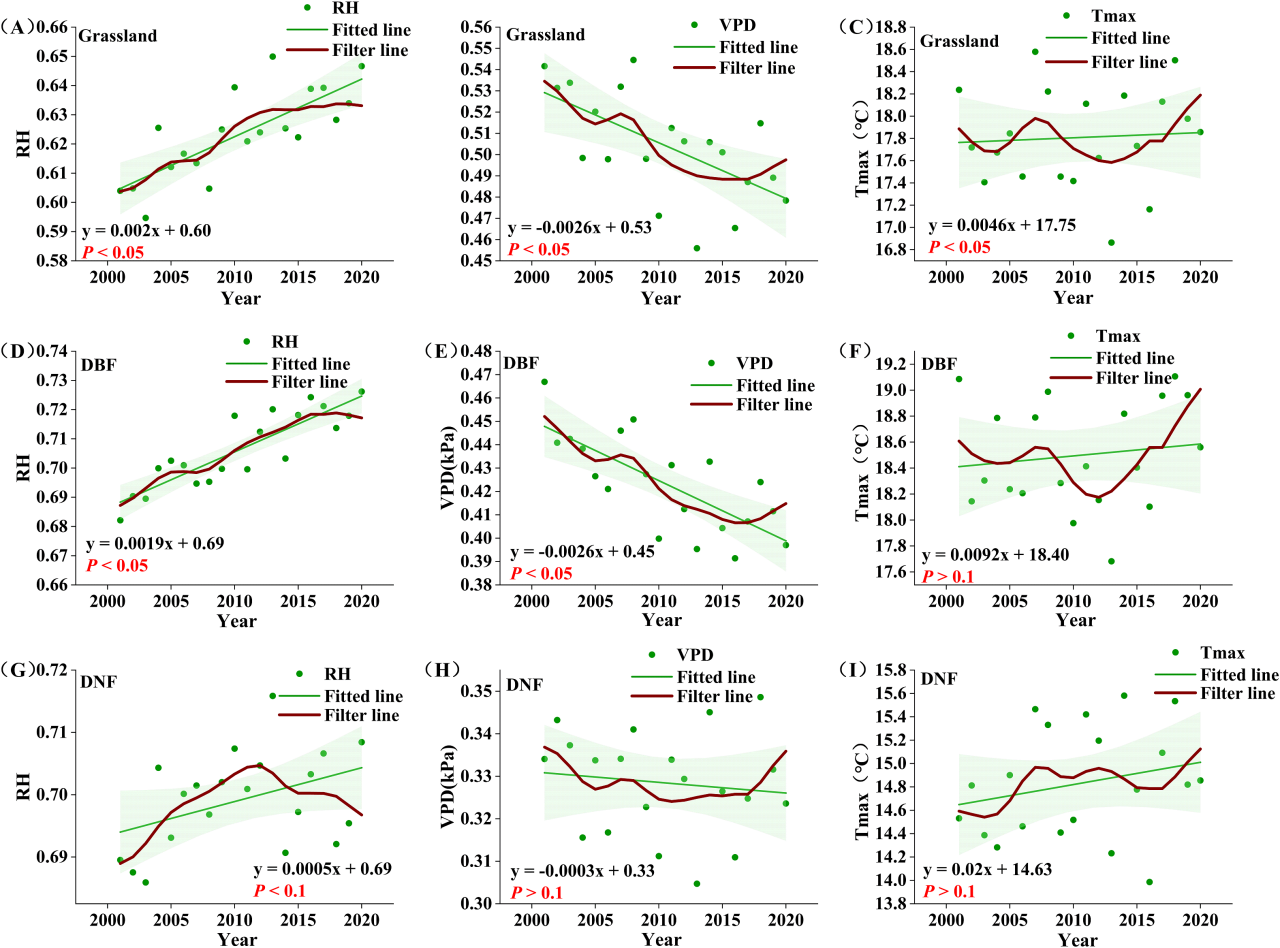
Growing season average climate factors for different vegetation types from 2001 to 2020. **(A-C)** : grassland RH, VPD, Tmax; **(D-F)** : DBF RH, VPD, Tmax; **(G-I)** : DNF RH, VPD, Tmax. The red line represents the result of nine-point binomial filter with reflected ends; the green line is the trend line.

According to the correlation results, RH, VPD, and T_max_ are most likely the climate factors that lead to the rise of GPP_GS_ before 2014 and the divergence after 2014. Comparing the temporal change characteristics of climate and GPP_GS_, apart from the overall trend, similarly, a 9-point binomial filtering method was used to reveal the longer-term change. As shown in **Figure 7**, T_max_ showed a nonsignificant increasing trend in all three vegetation types (*P*>0.1); VPD showed a significant decreasing trend in grassland and DBF areas (*P*< 0.1), while RH showed a significant increasing trend in all vegetation types (*P*<0.1). In DNF areas, there was a significant increasing trend in RH in the relatively arid Region 1 (*P*<0.1), but a non-significant decreasing trend in the relatively wet Region 2 (*P >*0.1).

Comparing the temporal change in climate factors and GPP_GS_ for each vegetation type, we see that T_max_ was consistent with the change in GPP_GS_ of all vegetation types before 2008. After 2008, T_max_ experienced a decrease and then an increase, at odds with steady increase in GPP_GS_ before 2014 and divergence after 2014. Spatially, the significant decreasing trend of T_max_ was mainly distributed in the northern part of the DBF distribution; the significant increasing trend of GPP_GS_ is also mainly distributed in the northern part of DBF. As GPP_GS_ is positively correlated with T_max_, changes in T_max_ could not be the cause.

For both grassland and DBF, VPD shifted from a sustained decline to an obvious increase around 2016, which clearly does not correspond with the temporal change in GPP_GS_. For DNF, there was a double peak in the interannual variability of VPD and neither was in 2014. Thus, VPD also could not be the cause of the transition of GPP_GS_ around 2014.

On the other hand, the temporal changes of RH were consistent with the temporal change in GPP_GS_ for all vegetation type. Grassland RH showed an obvious change point around in 2014, with a significant increasing trend before 2014 (*P*<0.05); the growth rate then declined and flattened after 2014, which is compatible with the main interannual change characteristics of grassland GPP_GS_. DBF RH basically maintained an upward trend, basically tracking the interannual change characteristics of DBF GPP_GS_, while DNF RH, on the other hand, generally matched the change pattern of DNF GPP_GS_, but with a lag of around one year. We note that the portion of the study area with the significant increase in RH generally overlapped with the region of significant increase in GPP (**Figures 2**, **6**).

In order to see whether there is a lag effect of climate factors on GPP_GS_, we calculated the correlation between GPP_GS_ and the previous year’s growing season climate indicators for the three vegetation types (**Table 3**). We found that the temperature of the previous year was not significantly correlated with the GPP_GS_. However, DBF and grassland GPP_GS_ were significantly positively correlated with the precipitation of the previous year and were significantly negatively correlated with the PAR of the previous year—more so than with the precipitation or PAR of the current year. The GPP_GS_ of all three vegetation types were significantly positively correlated with the RH and VPD of the previous year, again with higher correlations than with the RH and VPD of the current year. For DNF, GPP_GS_ in the relatively humid area (Region 2) also maintained significant relationships with previous year’s RH, VPD, and SM, while in the relatively arid area (Region 1) GPP_GS_ was only significantly positively correlated with previous year’s precipitation and, to a much lesser extent than in Region 2, RH. This suggests that the variation of GPP_GS_ in the study area is strongly influenced by the atmospheric moisture conditions of the previous year, while drier air in the previous year has an inhibitory effect on GPP_GS_. Grassland and DBF GPP_GS_ are also affected by the legacy of precipitation and solar radiation in the previous year.

**Table 3:** Correlation coefficients (r) between GPP_GS_ and climate indicators of the previous year’s growing season.

Region/Type	T_max_	T_min_	P	PAR	RH	VPD	SM
Grassland	-0.35	-0.19	0.54**	-0.44*	0.73***	-0.72***	0.33
DBF	-0.04	0.27	0.54**	-0.48**	0.81***	-0.70***	0.27
DNF	-0.22	-0.17	0.27	-0.08	0.75***	-0.66***	-0.15
Region1	-0.25	0.1	0.45*	-0.35	0.39*	-0.33	-0.12
Region2	-0.19	-0.13	0.12	0.01	0.69***	-0.65***	-0.06

*Significant at the 0.1 level; **Significant at the 0.05 level; ***Significant at the 0.01 level. Region1 represent DNF located in the relatively arid area, Region2 represent DNF located in the relatively humid area.

Comparing the possible influences of RH and VPD on GPP, we note that the temporal and spatial trends of VPD differ from those of GPP, whereas both the temporal and spatial changes in RH correspond with GPP_GS_ for each of the vegetation types. In addition, the correlations between space and regional average mean GPP_GS_ and the climate indicators were highly consistent, and RH in the previous year general had a stronger effect on GPP_GS_ than the current year for all three vegetation types. These is more obvious for DNF: DNF GPP_GS_ is not significantly correlated with the current year’s RH; however, it is strongly significantly correlated with the RH of the previous year. Spatially, the percentage of pixel points significantly correlated with the RH of the previous year is 19.63 percentage higher than that of the current year (**Tables 2**-**4**). Therefore, we conclude that the dominant factor causing the stagnation of GPP_GS_ in grassland and DNF after 2014 was RH, and the effect of RH on vegetation GPP_GS_ had a lagged effect. Thus, based on the spatial correspondence, temporal synchrony, and significance of the change, we argue that the change in GPP_GS_ in the study area during these two decades was mainly driven by RH, particularly the previous year’s RH.

**Table 4:** Percentage of pixels with positive correlation between GPP_GS_ and Relative Humidity (RH) of previous year and current year for different vegetation types.

vegetation type climatic factor	DNF	DBF	Grassland
Previous year	88.29%(29.76%)	85.92%(34.82%)	94.05%(41.71%)
Current year	49.67%(10.13%)	72.74%(34.46%)	91.00%(47.05%)

Percentage with significant correlations are shown in parentheses (*P*<0.05).

In addition, in order to determine whether the legacy effect of RH comes from the whole growing season or a specific period, we calculated the correlation between average RH in different periods of the previous year’s growing season (July-October, August-October, and September-October) and GPP for each vegetation type. We found that the strongest correlation between the GPP and the RH for these three periods was August-October (DNF: 0.60; DBF: 0.80; grassland: 0.72), but still slightly lower than the correlation between GPP and previous year’s entire growing season RH. This shows that the legacy effect of RH comes from the entire growing season, not just the last few months. To further validate the optimal lag period, we performed a cross-correlation function (CCF) analysis test and found results slightly lower than those for pairwise correlations, but the results were consistent with DNF, DBF, and grassland GPP_GS_ all having the strongest correlations with the previous year’s RH (0.72, 0.73, 0.67) and still stronger than with the current year’s RH (-0.07, 0.63, 0.62).

## 4 Discussion

### 4.1 Characteristics of GPP_GS_ changes in different vegetation types and their response to climate

Moisture and temperature and their synergistic effects are the main factors affecting vegetation growth and distribution (Sun et al., 2002). Overall, the spatial variation of vegetation GPP was highly consistent with the spatial variation of average hydrothermal conditions. The overall multiyear average GPP_GS_ in the study area showed a gradual increase from northwest to southeast with the increase of temperatures and moisture conditions, and the area with increasing GPP (85.46%) was much higher than that with decreasing GPP, corresponding with the overall trend toward increasing carbon sequestration capacity in China in recent years (Huang et al., 2022; Wu et al., 2023).

The mean GPP_GS_ of different vegetation types showed obvious differences: DBF displayed the largest GPP_GS_, mainly distributed in the temperate humid southeastern part of the study area; DNF GPP_GS_ was lower than DBF and mainly distributed in the colder northern part of the study area; and grassland GPP_GS_, lowest among the three vegetation types, was mainly distributed in temperate western part of study area. Previous studies have confirmed that these differences are closely related to the background climatic conditions, and the physiological structure of the vegetation (Liu et al., 2020a; Gui et al., 2021). On the one hand, the broad-leaved forests, with larger leaf area, have a strong carbon sink ability (Zhang et al., 2020; Ali et al., 2023); on the other hand, forests with a higher canopy structure and a deeper distribution of the root system are obviously more advantageous than the grasslands in terms of their carbon sequestration capacity (Ye et al., 2021; Lu and Yan, 2023).

We mainly considered climate change during the plant growing season when analyzing the effect of climate on vegetation GPP. The results showed that the average GPP_GS_ of different vegetation types vary in their sensitivity to climate indicators, which was consistent with the results of a previous study on the relationship between productivity and climate factors for grassland, shrublands, forests, etc (Xu et al., 2024). Our results further confirm that tree leaf form affects the sensitivity of GPP to climate. We found that DNF GPP is significantly correlated with current year T_max_ and PAR. This was explained by the facts that, on the one hand, because the DNF was distributed in the cold temperate climatic zone, PAR was significantly lower than that of other vegetation type distribution, so carbon absorption is more limited by light; on the other hand, conifer leaf area is relatively small and mainly receives scattered light, resulting in a large effect of light on its assimilation process (Chen et al., 2024a).

Grassland and DBF GPP_GS_ are mainly affected by changes in RH, which integrates the water and heat conditions of a region (Niu et al., 2020). RH has been found to be a dominant factor influencing the spatial differentiation of biomass in vegetation (Dragoni et al., 2011). We suggest that the role of RH in determining the changes in vegetation productivity can be explained in the following ways: When RH is high, atmospheric water vapor is more likely to condense into water droplets and fall on the ground to replenish soil moisture, which in turn reduces evaporation loss of water, reduces the need to transport water from the soil through the roots, and slows down the transpiration process, which effectively reduces the dissipation of moisture from the vegetation, keeps the vegetation in a better water status, and promotes photosynthesis (Baldocchi et al., 2018). When RH is low, it contributes to an increase in transpiration rate, inducing rapid closure of leaf stomata to reduce excessive water loss, however, this also results in the inaccessibility of carbon dioxide, which depletes carbohydrate reserves and leads to carbohydrate starvation at the tissue level, thus inhibiting GPP growth (Buckley, 2019; Hsu et al., 2021; Song et al., 2021). The fact that air moisture emerges as the dominant factor for recent GPP change implies that relative importance in water conditions will increase under conditions of global warming.

Unlike the results of previous studies that found that changes in grassland biomass were more sensitive to precipitation than to temperature (Beer et al., 2010), the present study found that grassland GPP_GS_ was not significantly correlated with precipitation or temperature, which may be due to the distribution of grassland in the study area. Grasslands selected for the present study were mainly located in the northern part of the study area where precipitation varied considerably; this led to a polarization of the correlation between grassland GPP_GS_ and precipitation, with the proportion of image points with positive and negative significant correlations between GPP_GS_ and precipitation accounting for 8.85% and 9.50%, respectively (**Figure 6**). These nearly equal proportions neutralized the relationship of grassland GPP_GS_ with precipitation, which ultimately led to a nonsignificant correlation.

In contrast to grassland GPP_GS_, both forest vegetation types were regulated by precipitation, but they showed opposite significant correlations: DNF is significantly negatively correlated with precipitation while DBF was significantly positively correlated with precipitation. This may be due to the fact that the distribution of DNF in the study area is mainly in the eastern coastal region of Russia, which is subject to the influence of the southeast monsoon from the sea in summer and receives more abundant precipitation. Further more, deciduous-coniferous forests are inherently resistant to cold and drought and do not have a high water demand (Jia et al., 2023). Therefore, increased precipitation inhibited the accumulation of GPP in relative humid DNF distribution.

The same is true for soil moisture. The dominant DNF species in the study area, the Dahurian larch (*Larix gmelinii*), is adapted to survive in well-drained soils (Rehfeldt and Jaquish, 2010). As an abundance of precipitation raises soil moisture and can lead to water-filled soil pores, root hypoxia, the inhibition of respiration and metabolic activity of aerobic microorganisms in tree roots, and a reduction of water and nutrient uptake efficiency, thus limiting photosynthesis (Costa et al., 2023; Peng et al., 2024). This is consistent with the significant negative correlation between GPP and SM in the relatively humid DNF distribution. In addition, the effect of precipitation on vegetation GPP is generally less than that of relative humidity, because precipitation is not directly converted into available water for vegetation, but indirectly regulate the carbon sequestration capacity of vegetation through affects soil and atmospheric humidity (Driesen et al., 2020). For vegetation growing in low-latitude south China, prior research found that relative humidity strongly affects GPP, surpassing the effects of precipitation and soil moisture (Xu et al., 2024), consistent with our results. However, that study observed lag effects in net solar radiation and surface temperature, while our results confirm that RH had the strongest legacy effects on vegetation in middle to high latitude vegetation.

It is noteworthy that the GPP_GS_ of all vegetation types is affected more by the RH of the previous year than by the RH of the current year, DNF most of all. This indicates that the degree of atmospheric drought in the previous year would largely affect carbon absorption in the current year. This is related with to legacy effect of changes in the moisture available at various points in the vegetation life cycle: One possibility is that changes in air humidity in the previous year may alter plant roots and leaves development and density (Liu et al., 2021a; Hu et al., 2025), which in turn affects vegetation carbon absorption in the coming year. This effect is common in arid to semi-moist climate ecosystems: the strong legacy effect of previous year’s precipitation on NPP leads to improved moisture conditions, enhancing root and canopy development, which is then helpful for the next year’s carbon absorption (Sala et al., 2012).

In addition, trade-offs between immediate plant growth and long-term survival may also be considered (Chuine, 2010; Yuan et al., 2019). In terms of long-term climate adaptation, photosynthetic products from the growing season of the previous year may not be used for immediate growth, but, rather, stored as non-structural carbohydrates in the root system, branches, or seeds (Meng et al., 2023a), which are mainly used to cope with climatic stress and for initiating growth early in the following year. Thus, carbon storage for survival is prioritized over immediate growth to reduce the risk of mortality (Olano et al., 2023), it seems that improvement in hydro-thermal condition can reduce the survival cost which is benefit for plant growth.

Our results highlight how GPP responses to climate vary considerably among different vegetation types and support prior findings that time-lag effects are quite important for better predicting and evaluating changes in vegetation dynamics that may accompany global warming (Wu et al., 2015).

### 4.2 Characteristics of GPP_GS_ response to phenology for different vegetation types

Among the relationships between phenology and GPP_GS_, the significant negative correlation between vegetation GPP_GS_ and POS—strongest in grasslands—is most notable. This relationship can be explained in two ways. First, earlier POS promotes vegetation carbon assimilation throughout the growing season, which ultimately leads to an increase in productivity (Wang and Wu, 2019). Second, plants experiencing earlier POS can take advantage of favorable spring climatic conditions (Gonsamo et al., 2018; Wang and Wu, 2019), enhance photosynthesis, and mitigate to some extent the potential damage to plant growth caused by high summer temperatures and drought (Liu et al., 2021b), prolonging the growing season and ultimately increasing vegetation productivity. Grasslands have more rapid growth rates and resource acquisition capacity compared to forests (Yan et al., 2025), which means that more of their productivity is concentrated in the peak growth period when temperature and moisture conditions are favorable. The peak growth period inherently represents the time of maximum availability of resources for vegetation growth (Shen et al., 2024a); favorable climatic conditions contribute to the fact that the period in which the POS occurs accounts for more than half of the year’s productivity (Yang et al., 2024). Furthermore, the shorter growth period of herbaceous plants also accentuates the role of changes in the summer peak growth period in influencing productivity (Zhou, 2020).

Overall, the relationship between GPP_GS_ changes and phenological responses was not as strong as that with climatic factors during 2001-2020. On the one hand, because the 2001–2020 POS showed a non-significant trend of advancement in all vegetation types, its contribution to the GPP change was necessarily limited. On the other hand, because the study area is located in the middle to high latitudes of the Northern Hemisphere, which is quite sensitive to climate change, GPP is mainly affected by the efficiency of photosynthesis, which is directly dependent on climatic factors such as temperature, precipitation, and so on (Du et al., 2022; Wang et al., 2022b). While phenological changes are often attributed to the corresponding climate change effects (Coomes et al., 2014), their independent effects may be masked by concurrent climate changes. In addition, it has been found that for many northern ecosystems, the benefits of warmer springs on growing-season ecosystem productivity are effectively compensated by the accumulation of seasonal water deficits (Buermann et al., 2018), i.e., as the phenology advances, GPP is likely to become more strongly influenced by moisture conditions.

Our results emphasize that the capacity of middle to high latitude Northeast Asia vegetation to act as a future carbon sink depends critically on the air moisture. The contribution of water deficits to GPP variation suggests that carbon uptake in northern middle to high latitude ecosystems may be more determined by moisture conditions. This can help provide an important basis for ecosystem management or carbon sink policies under future climate scenarios. Ecosystem managers may need to focus on maintaining soil moisture through enhanced water retention strategies, such as improving organic matter content and implementing water-conserving land use practices. Management decisions should consider not just current conditions but also how previous years’ climate patterns will influence ecosystem responses. This may require developing early warning systems and adaptive management protocols that can respond to multi-year climate patterns.

## 5 Conclusions

In this study, we analyzed the relationships between vegetation growing season cumulative gross primary productivity (GPP_GS_) and vegetation phenology and climatic factors in the mid- to high-latitude regions of East Asia to assess the dominant factors influencing the changes in GPP_GS_ of different vegetation types from 2001 to 2020. We found:

1. Among the phenology factors, the advancement of POS has a positive effect on GPP_GS_ of all three vegetation types, with grassland being the most affected by POS due to its strong opportunist characteristics; the advancement of SOS also has a positive effect on GPP_GS_ of grasslands and DBF, while DNF GPP_GS_ was less responsive to phenology variation.
2. Relative humidity in the previous year had a stronger positive effect than the current year on changes in the GPP_GS_ of all three vegetation types, especially that of DNF in humid areas. Soil moisture of current year has negative effect on the vegetation productivity of DNF, and T_max_ and PAR in the current year have positive effect on the vegetation productivity of DNF. For both grasslands and DNF, the vegetation in drier regions had a higher sensitivity to precipitation.
3. Over these two decades, the overall trend of vegetation GPP_GS_ in the middle and high latitude regions of East Asia showed a significant increase, with obvious differences for different vegetation types. Before 2014, all three vegetation types showed significant increasing trends. Since 2014, DBF GPP_GS_ maintained its increase trend, while the growth rate of grassland GPP_GS_ flattened and that of DNF slightly decreased. RH dominate the changes of GPP_GS_ for all three vegetation types, indicating that changes in vegetation productivity were more influenced by atmospheric moisture conditions in this study area during recent decades.

In conclusion, this paper summarizes the effects of climatic and phenological changes on vegetation GPP_GS_, highlighting the significant legacy effects of changes in RH of the previous year on GPP and pointing to the importance of changes in RH accompanying global warming. In addition, we find that DNF GPP_GS_ differs from the other vegetation types in that it is less affected by phenology changes, affected more by lower temperatures and excess moisture. At the same time, both grasslands and coniferous forests are also strongly influenced by background climate conditions. Therefore, we need to pay attention to the effects of changing climate and phenology on different vegetation types. These findings provide new insights into the complex relationships among phenology, climate and vegetation productivity.

## Data Availability

The original contributions presented in the study are included in the article/supplementary material. Further inquiries can be directed to the corresponding author.
